# Predictors of current tobacco smoking by adolescents in Nigeria: Interaction between school location and socioeconomic status

**DOI:** 10.18332/tid/117959

**Published:** 2020-03-03

**Authors:** Ijeoma U. Itanyi, Chika N. Onwasigwe, Deborah Ossip, Benjamin S. C. Uzochukwu, Scott McIntosh, Emmanuel N. Aguwa, Sijiu Wang, Chima A. Onoka, Echezona E. Ezeanolue

**Affiliations:** 1 Department of Community Medicine, University of Nigeria, Enugu, Nigeria; 2 Department of Community Medicine, University of Nigeria Teaching Hospital, Enugu, Nigeria; 3 Department of Public Health Sciences, University of Rochester, Rochester, United States; 4 Institute of Maternal and Child Health, University of Nigeria, Enugu, Nigeria

**Keywords:** tobacco smoking, adolescent, predictors, interaction, Nigeria

## Abstract

**INTRODUCTION:**

Tobacco smoking is the largest preventable cause of global mortality, with its prevalence increasing in Sub-Saharan Africa, particularly among adolescents. We sought to determine the factors associated with tobacco smoking among Nigerian school adolescents and investigate the interaction between school location and socioeconomic status (SES).

**METHODS:**

Using a cross-sectional study design, 4332 eighth to tenth grade students in rural and urban secondary schools in Enugu State, Nigeria, were selected by stratified two-stage cluster sampling. We collected data using a modified Global Youth Tobacco Survey (GYTS) Core Questionnaire. Outcome measures were current smoking of cigarettes and other smoked tobacco. Multilevel mixed effects logistic regression models were used to determine factors associated with current tobacco smoking and were considered statistically significant at p<0.05.

**RESULTS:**

Prevalences of current smoking of cigarettes and other smoked tobacco were 13.3% (95% CI: 11.3–15.7) and 5.8% (95% CI: 4.6–7.2), respectively. Possession of higher weekly allowance, exposure to secondhand smoke or tobacco advertisements, having smoking parents, friends or classmates who smoke, and sale of cigarettes near school, were positively associated with current smoking of tobacco. Female sex, having both parents employed and being exposed to tobacco teaching in school were negatively associated with current cigarette smoking while increasing age and high father’s SES were negatively associated with current smoking of other tobacco products. There was an interaction between school location and father’s SES in the association with cigarette smoking. The higher odds of smoking in rural versus urban schools were much higher for students with fathers of high SES compared to low SES. In rural schools, high SES was associated with higher odds of smoking, but in urban schools low SES was associated with higher odds of smoking. **CONCLUSIONS** Environmental factors are associated with adolescent tobacco smoking. Tobacco control programs should use targeted strategies that vary depending on the local context.

## INTRODUCTION

Tobacco use is the largest preventable cause of death globally, and is responsible for more than 8 million deaths per year^1^. Most developed countries have recorded a decreasing prevalence of tobacco smoking; however, the prevalence has been increasing in low- and middle-income countries, particularly Sub-Saharan Africa, partly due to cigarette affordability and aggressive marketing by tobacco companies^2^. In Nigeria, about 16100 tobacco-related deaths occur annually^3^. It is likely that these numbers may be grossly underestimated because of weak surveillance systems. In addition, 5.6% (4.7 million) of Nigerian adults currently use a tobacco product and 3.9% (3.1 million) adults are current tobacco smokers^4^. Of greater concern is tobacco smoking by children and adolescents where 25000 Nigerian children (aged 10–14 years) smoke cigarettes each day^3^. Cigarettes are affordable for young people in Nigeria because they are still being sold in single sticks, despite the provision of Article 16 of the Framework Convention on Tobacco Control to which Nigeria is a signatory^5^. The dangers of tobacco smoking from an early age are well established and include diseases of the heart, respiratory system, central nervous system and cancers of almost all organs of the body. There is also an increased risk of addiction to nicotine. The dangerous effects of tobacco smoking on the developing brains of children and adolescents^6^ are of great concern, necessitating more effective tobacco control globally.

Several factors have been shown to influence tobacco smoking by adolescents^7-13^. These include sociodemographic, environmental, and psychosocial factors. A recent systematic review of studies on adolescent tobacco smoking in Nigeria showed peer smoking, parental smoking, media advertisements, male gender, increasing age, low parental education, and family conditions as significant determinants of tobacco smoking^14^. Most of these studies were school-based, yet none investigated the association between school geographical location or socioeconomic status (SES) and adolescent tobacco smoking. These are important contextual factors that have been shown to influence adolescent tobacco use^7-13^. Understanding the association between these contextual factors and adolescent tobacco smoking is necessary for developing effective smoking prevention and cessation interventions. Moreover, it is likely that tobacco smoking could vary between schools but this has not been investigated in other studies in Nigeria.

The objectives of this study were to determine the factors associated with adolescent tobacco smoking in Nigeria and investigate the interaction between school location and socioeconomic status (SES). To the best of our knowledge, this is the first study to investigate determinants of adolescent tobacco smoking in Nigeria using multilevel analysis.

## METHODS

### Study design, participants and procedure

Details of the study design and procedure have been described elsewhere^15^. In brief, this study was carried out in urban and rural secondary schools in Enugu State, southeastern Nigeria, using the Global Youth Tobacco Survey (GYTS) design. Eligible participants were students in Junior Secondary 2 and 3, and Senior Secondary 1 (i.e. JS2, JS3, SS1) corresponding to 8th, 9th and 10th grade. Stratified two-stage cluster sampling was used to select 25 schools (first stage) and classes (second stage) independently in urban and rural locations using systematic sampling at each stage. A sample of 80 students was sought in each school, corresponding to 2000 students per stratum. A pretested self-administered questionnaire (Supplementary file) adapted from GYTS Core Questionnaire^15,16^ was used to collect data without identifiers, from November to December, 2015.

The Health Research Ethics Committee of the University of Nigeria Teaching Hospital (NHREC/05/01/2008B-FWA000024581RB00002323) provided ethical approval for the study. We also obtained approval from the Ministry of Education and principals of the selected schools. The students gave written assent and principals of selected schools acted as legal guardians of the students and provided written consent, as was done in previous GYTS studies in Nigeria.

### Measures

There were two outcome measures: 1) current cigarette smoking, and 2) current smoking of other tobacco products. We defined current cigarette smoking as use on one or more days within past 30 days. Current smoking of other tobacco products (cigars, pipes, shisha, bidis) was defined as any use within past 30 days.

Sociodemographic characteristics included age group (10–12, 13–15 and 16–19 years); sex (male/female); grade (JS2/JS3/SS1); students’ status (day student/boarder); possession of weekly spending money (none, ≤100 NGN or about 0.27 US$, >100 NGN); parents’ work status (initially measured in four categories of neither/father only/mother only/both parents, but father only and mother only were combined into one parent); parents living together (yes/no); and SES, which was measured using parental education (low SES for parental education of secondary or lower/high SES for tertiary parental education). SES was determined for each parent, and was treated as both an exposure variable and an effect modifier. School characteristics included geographical location (urban/rural) and school type (public/private). A rate of 1 US$ to 360 NGN was used for currency conversion.

Environmental factors included exposure to secondhand tobacco smoke (SHS) (‘yes’ if exposed at home, indoor or outdoor public places in last 7 days, ‘no’ if unexposed); exposure to pro-tobacco advertisements (‘yes’ if exposed to tobacco promotions/advertisements at points-of-sale or watching use of tobacco on TV in past 30 days or possess an item with tobacco logo on it or offered a free tobacco product, ‘no’ if unexposed); exposure to anti-tobacco messages on media, at events/gatherings, at home, or by health warnings on cigarette packages in past 30 days (yes/no); inclusion of tobacco in school curriculum (‘yes’, if within past 12 months, the student was taught in class about dangers of tobacco, read about health effects of tobacco in school books, or discussed in class the reasons why adolescents smoke, ‘no’ if student did not receive any tobacco teaching in school); sale of cigarettes near school (‘yes/no/don’t know’ but ‘don’t know’ was combined with ‘no’) was used to measure tobacco access and availability; peer tobacco use was assessed with friends’ smoking and classmates’ smoking, each categorized as none/some/most/all; and parental smoking (none/one parent/both parents).

### Statistical analyses

**Figure 1:**
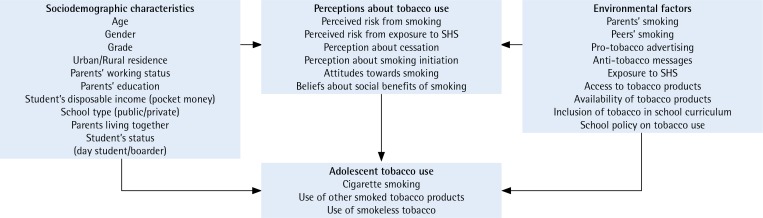
Conceptual framework showing factors influencing adolescent tobacco use

Data were analyzed using Stata Version 11. We computed weighted prevalence estimates and 95% confidence intervals for each type of tobacco smoking. Multilevel mixed effects logistic regression models were used to determine predictors of each outcome – current cigarette smoking and current smoking of other tobacco product. We started with a 2-level null model that contained each of the outcomes with students nested within schools and a random intercept at school level, assuming covariance ‘identity’ structure, the default method used when only one variable is specified in the random part of the model. Each covariate was then added to the fixed part of the model while maintaining random intercept at school level. The covariates examined included sociodemographic characteristics, school characteristics, and environmental factors. Covariates that reached statistical significance of ≤0.2 in the bivariable analyses and those specified a priori were included in the multivariable model with random intercept at school level using forward selection^17^. A conceptual framework (Figure 1) guided the multivariable analysis.

We examined if the odds of smoking varied by geographical location by introducing random coefficient at location. Likelihood ratio test comparing models with and without random coefficient was in favor of the simpler model without random coefficient at location. To test whether SES modified the smoking–school location association, an interaction term between school location and SES was introduced in the multivariable models. A separate model was developed for fathers’ SES and mothers’ SES.

Each of the models with the interaction term was compared to the model without the interaction term using a likelihood ratio test. Mothers’ SES showed no association with adolescent smoking, so models with fathers’ SES were used. There was no evidence of interaction in the model for current smoking of other tobacco products. The final model for predicting current cigarette smoking did not include students’ grade, students’ status, and school type. Similarly, grade, students’ status and inclusion of tobacco in school curriculum were not included in the final model for predicting current smoking of other tobacco. We considered p<0.05 as statistically significant and restricted analyses to adolescents.

## RESULTS

There were 4332 adolescents out of 4354 respondents, with 2230 and 2102 students from urban and rural schools, respectively. Response rates were 84.4% in urban and 80.6% in rural locations. Students in urban schools were younger, more of the parents were employed and lived together, and had a higher proportion of parents with high SES (Table 1). Reported prevalence of current smoking of cigarettes

**Table 1:** Characteristics of adolescents in secondary schools in Enugu State

*Variables*	*Total (N=4332) n (%)*	*Urban (n=2230) n (%)*	*Rural (n=2102) n (%)*	*F*^a^	*p*
**Currently smokes cigarettes**					
Yes	575 (13.3)	225 (10.1)	350 (16.7)	10.3	0.002
No	3757 (86.7)	2005 (89.9)	1752 (83.3)		
**Currently smokes other tobacco**					
Yes	254 (5.8)	90 (4.0)	164 (7.6)	10.1	0.003
No	4078 (94.2)	2140 (96.0)	1938 (92.4)		
**Age** (years)					
10–12	430 (9.8)	268 (11.8)	162 (7.7)	4.25	0.025
13–15	2992 (68.9)	1587 (80.0)	1405 (66.7)		
16–19	910 (21.3)	375 (17.2)	535 (25.6)		
**Sex**					
Female	2443 (56.3)	1256 (56.1)	1187 (56.4)	0.002	0.962
Male	1889 (43.7)	974 (43.9)	915 (43.6)		
**Grade**					
JS 2	1216 (28.2)	522 (22.8)	694 (33.9)	1.79	0.173
JS 3	1567 (35.7)	938 (41.6)	629 (29.5)		
SS 1	1549 (36.1)	770 (35.5)	779 (36.6)		
**Student status**					
Day student	3796 (87.7)	1824 (81.8)	1972 (93.9)	3.15	0.083
Boarder	536 (12.3)	406 (18.2)	130 (6.1)		
**School type**					
Public	2906 (66.1)	1637 (73.9)	1269 (57.9)	1.53	0.223
Private	1426 (33.9)	593 (26.1)	833 (42.1)		
**Weekly spending money** (NGN)					
None	1693 (38.8)	848 (38.0)	845 (39.6)	0.85	0.417
≤100	1731 (40.0)	872 (39.0)	859 (41.1)		
>100	908 (21.2)	510 (23.0)	398 (19.3)		
**Parents’ work status**					
None	245 (5.6)	81 (3.7)	164 (7.6)	14.76	<0.001
One parent	1184 (27.4)	499 (22.3)	685 (32.9)		
Both parents	2903 (67.0)	1650 (74.0)	1253 (59.5)		
**Parents live together**					
Yes	3712 (85.5)	1978 (88.7)	1734 (82.1)	15.2	<0.001
No	620 (14.5)	252 (11.3)	368 (17.9)		
**Father’s SES**					
Low	2984 (69.3)	1341 (60.4)	1643 (78.6)	14.01	<0.001
High	1348 (30.7)	889 (39.6)	459 (21.4)		
**Mother’s SES**					
Low	3094 (71.8)	1375 (62.0)	1719 (82.0)	14.6	<0.001
High	1238 (28.2)	855 (38.0)	383 (18.0)		
**Exposure to secondhand smoke**					
Yes	2627 (60.4)	1379 (61.7)	1248 (59.1)	0.79	0.380
No	1705 (39.6)	851 (38.3)	854 (40.9)		
**Exposure to anti-tobacco messages**					
Yes	3501 (80.8)	1860 (83.4)	1641 (78.0)	5.39	0.025
No	831 (19.2)	370 (16.6)	461 (22.0)		
**Taught about tobacco in school**					
Yes	3206 (74.0)	1744 (78.1)	1462 (69.7)	10.28	0.002
No	1126 (26.0)	486 (21.9)	640 (30.3)		
**Exposed to tobacco advertisements**					
Yes	3155 (72.9)	1639 (73.6)	1516 (72.2)	0.352	0.555
No	1177 (27.1)	591 (26.4)	586 (27.8)		
**Have smoking parents**					
None	3884 (89.6)	2046 (91.7)	1838 (87.4)	6.81	0.002
One parent	333 (7.7)	151 (6.8)	182 (8.5)		
Both parents	115 (2.7)	33 (1.5)	82 (4.1)		
**Have smoking friends**					
None	3719 (85.8)	1980 (88.7)	1739 (82.7)	8.07	<0.001
Some	469 (10.9)	193 (8.8)	276 (13.2)		
Most	75 (1.7)	37 (1.6)	38 (1.7)		
All	69 (1.6)	20 (0.9)	49 (2.4)		
**Have smoking classmates**					
None	3521 (81.0)	1859 (83.0)	1662 (78.9)	1.93	0.139
Some	610 (14.4)	278 (12.8)	332 (16.0)		
Most	147 (3.4)	70 (3.2)	77 (3.7)		
All	54 (1.2)	23 (1.0)	31 (1.4)		
**Sale of cigarettes near school**					
Yes	559 (12.8)	275 (12.4)	284 (13.3)	0.16	0.690
No	3773 (87.2)	1955 (87.6)	1818 (86.7)		

a Design-based χ^2^.

NGN: Nigerian Naira, 100 NGN about 0.27 US$. JS: junior secondary school level. SS: senior secondary school level.

and other smoked tobacco were 13.3% (95% CI: 11.3–smoking. Females were less likely to be current 15.7) and 5.8% (95% CI: 4.6–7.2), respectively.

Table 2 shows predictors of current cigarette smoking. Females were less likely to be current smokers of cigarettes (OR=0.73; 95% CI: 0.59–0.91). Possession of weekly spending money of more than 100 NGN (about 0.27 US$) increased the odds of cigarette smoking by 1.7 (95% CI: 1.29–2.17). There was a reduction in odds of cigarette smoking when parents were employed, with 42% reduction in odds when both parents were employed. Students who were exposed to secondhand smoke (OR=2.01; 95% CI: 1.59–2.54) or to tobacco advertisements (OR=1.39; 95% CI: 1.07–1.80) had higher odds of smoking cigarettes. Inclusion of tobacco in school curriculum reduced the odds in cigarette smoking by 41%. There was a graded increase in odds of cigarette smoking as the number of parent smokers or peer smokers increased, with friends’ smoking having the greatest effect (if ‘all’ of friends smoked). Students who could buy cigarettes near their school had 1.8 times higher odds of being current smokers.

**Table 2:** Predictors of current cigarette smoking among adolescents in secondary schools in Enugu State

*Variables*	*Crude*				*Adjusted*		

	*OR*	*95% CI*	*p*	*OR*	*95% CI*	*p*
**Geographical location**						
Urban	1			1		
Rural	1.77	1.24–2.51	0.002	1.23	0.86–1.77	0.264
**Age** (years)				1		
10–12	1					
13–15	0.96	0.70–1.33	0.805	0.76	0.54–1.07	0.117
16–19	1.26	0.88–1.80	0.216	0.75	0.51–1.11	0.15
**Sex**						
Female	0.6	0.49–0.74	<0.001	0.73	0.59–0.91	0.005
Male	1			1		
**Weekly spending money** (NGN)						
None	1			1		
≤100	1.25	1.01–1.55	0.043	1.1	0.88–1.39	0.405
>100	2.13	1.67–2.71	<0.001	1.67	1.29–2.17	<0.001
**Parents’ work status**						
None	1			1		
One parent	0.89	0.62–1.26	0.499	0.95	0.65–1.41	0.815
Both parents	0.5	0.35–0.70	<0.001	0.58	0.39–0.86	0.006
**Parents live together**						
Yes	1			1		
No	1.59	1.26–2.01	0<0.001	1.26	0.97–1.63	0.08
**Father’s SES**						
Low	1			1		
High	0.94	0.76–1.17	0.58	0.77	0.55–1.06	0.111
**Exposure to secondhand smoke**						
Yes	2.44	1.98–3.01	<0.001	2.01	1.59–2.54	<0.001
No	1			1		
**Exposure to anti-tobacco messages**						
Yes	1			1		
No	1.32	1.04–1.68	0.023	1.12	0.84–1.48	0.438
**Taught about tobacco in school**						
Yes	0.73	0.60–0.88	0.001	0.59	0.47–0.75	<0.001
No	1			1		
**Exposed to tobacco advertisements**						
Yes	1.72	1.37–2.16	<0.001	1.39	1.07–1.80	0.013
No	1			1		
**Have smoking parents**						
None	1			1		
One parent	2.73	2.08–3.60	<0.001	1.51	1.10–2.08	0.01
Both parents	3.9	2.57–5.91	<0.001	2.26	1.43–3.57	<0.001
**Have smoking friends**						
None	1			1		
Some	3.33	2.61–4.24	<0.001	2.06	1.56–2.71	<0.001
Most	6.08	3.71–9.96	<0.001	3.39	1.96–5.86	<0.001
All	19.75	11.41–34.18	<0.001	7.08	3.85–13.04	<0.001
**Have smoking classmates**						
None	1			1		
Some	2.16	1.70–2.73	<0.001	1.3	0.99–1.70	0.056
Most	3.76	2.55–5.53	<0.001	1.49	0.95–2.34	0.081
All	13.75	7.62–24.83	<0.001	4.56	2.31–9.00	<0.001
**Sale of cigarettes near school**						
Yes	2.49	1.98–3.13	<0.001	1.79	1.39–2.31	<0.001
No	1			1		
**Interaction between father’s SES and school location**						
Yes				1.86	1.17–2.94	0.008
No				1		

Null model variance (SE) = 0.356 (0.095). Full model variance (SE) = 0.225 (0.07). NGN: Nigerian Naira, 100 NGN about 0.27 US$.

A significant interaction was found between school location and father’s SES for cigarette smoking odds (OR for interaction term=1.86; 95% CI: 1.17–2.94; cigarette smokers (OR for interaction term=1.86; 95% CI: 1.17–2.94; p=0.008) (Table 2). Stratum-specific odds ratios for school location and father’s SES are presented in Table 3 as recommended by Knol et al.^18^. Compared to students of low SES in urban schools, students of high SES in rural schools had higher odds of being current cigarette smokers (OR=1.75; 95% CI: 1.14–2.69). The odds of current smoking in rural compared to urban schools were much higher for students of high SES compared to students of low SES (2.28 vs 1.23). Conversely, in urban schools, students of high SES had lower odds of being current smokers although not statistically significant (OR=0.77; 95% CI: 0.55–1.06) while in rural schools, high SES increased the odds of smoking (OR=1.42; 95% CI: 1.03–1.96).

**Table 3:** Interaction between school geographical location and socioeconomic status on odds of current cigarette smoking among adolescents in secondary schools in Enugu State

*Variable*	*Urban*			*Rural*			*FSL*		

	*AOR*	*95% CI*	*p*	*AOR*	*95% CI*	*p*	*AOR*	*95% CI*	*p*
Low SES	1			1.23	0.86–1.77	0.264	1.23	0.86–1.77	0.264
High SES	0.77	0.55–1.06	0.111	1.75	1.14–2.69	0.011	2.28	1.43–3.65	0.001
FSES	0.77	0.55–1.06	0.111	1.42	1.03–1.96	0.033			

FSES: For SES within strata of school location. FSL: for school location within strata of SES. Measure of effect modification on multiplicative scale: ratio of ORs (95% CI) = 1.86 (1.17–2.94); p=0.008. AOR: adjusted OR for age, sex, weekly spending money, parents work status, parents living together, exposure to secondhand tobacco smoke, anti-tobacco message or pro-tobacco advertisement, taught about tobacco in school, have smoking parents, friends or classmates, and sale of cigarettes near school.

Predictors of current smoking of other tobacco products are shown in Table 4. Students who attended rural schools (vs urban), had weekly spending money >100 NGN (vs none), were exposed to secondhand smoke or pro-tobacco advertisements, had smoking parents (both smoked), friends (some, most, or all) or classmates (all) or could buy cigarettes near school, were more likely to be current smokers of other tobacco products. Conversely, older students (vs 10–12 years age group), female students (vs males), and students of high SES (vs low SES) were less likely to be current smokers of other tobacco products. There was no evidence of interaction between school location and fathers’ SES (p=0.22).

**Table 4:** Predictors of current smoking of other tobacco products among adolescents in secondary schools in Enugu State

*Variables*	*Crude*			*Adjusted*		

	*OR*	*95% CI*	*p*	*OR*	*95% CI*	*p*
**Geographical location**						
Urban	1			1		
Rural	1.89	1.20–2.98	0.006	1.66	1.07–2.60	0.025
**School type**						
Public	1			1		
Private	0.63	0.37–1.07	0.087	0.64	0.40–1.04	0.069
**Age** (years)						
10–12	1			1		
13–15	0.74	0.49–1.14	0.17	0.59	0.38–0.91	0.017
16–19	1.04	0.65–1.69	0.858	0.64	0.38–1.05	0.077
**Sex**						
Female	0.55	0.41–0.73	<0.001	0.64	0.47–0.86	0.003
Male	1			1		
**Weekly spending money** (NGN)						
None	1			1		
≤100	1.16	0.85–1.58	0.354	1.04	0.76–1.44	0.799
>100	1.92	1.37–2.68	<0.001	1.71	1.20–2.43	0.003
**Parents’ work status**						
**None**	1			1		
One parent	1.1	0.66–1.82	0.719	1.12	0.66–1.90	0.678
Both parents	0.55	0.33–0.90	0.018	0.62	0.36–1.06	0.078
**Parents live together**						
Yes	1			1		
No	1.47	1.06–2.04	0.022	1.16	0.81–1.65	0.419
**Father’s SES**						
Low	1			1		
High	0.6	0.43–0.83	0.002	0.65	0.46–0.92	0.015
**Exposure to secondhand smoke**								
Yes	2.12	1.57–2.86	<0.001	1.6	1.16–2.21	0.004		
No	1			1				
**Exposure to anti-tobacco messages**								
Yes	1			1				
No	1.87	1.27–2.74	0.001	1.46	0.97–2.20	0.072		
**Exposed to tobacco advertisement**								
Yes	1.88	1.34–2.65	<0.001	1.47	1.02–2.13	0.04		
No	1			1				
**Have smoking parents**								
None	1			1				
One parent	2.46	1.89–5.47	1	1.42	0.92–2.17	0.112		
Both parents	3.22	1.68–3.60	<0.001	2.31	1.31–4.07	0.004		
**Have smoking friends**								
None	1			1				
Some	2.33	1.66–3.28	<0.001	1.7	1.15–2.51	0.008		
Most	3.13	1.58–6.18	0.001	2.22	1.05–4.69	0.036		
All	7.42	4.17–13.20	<0.001	3.2	1.61–6.34	0.001		
**Have smoking classmates**								
None	1			1				
Some	1.18	0.83–1.70	0.357	0.69	0.46–1.03	0.068		
Most	1.64	0.90–3.01	0.108	0.71	0.37–1.39	0.325		
All	6.3	3.31–11.98	<0.001	2.22	1.06–4.67	0.035		
**Sale of cigarettes near school**								
Yes	2.46	1.80–3.36	<0.001	1.87	1.34–2.61	<0.001		
No	1			1				

Null model variance (SE) = 0.520 (0.160). Full model variance (SE) = 0.322 (0.123). NGN: Nigerian Naira, 100 NGN about 0.27 US$.

The odds of both current cigarette smoking and current smoking of other tobacco products differed by school (p<0.001). Addition of sociodemographic, school-level and environmental factors to the null models reduced the variance in current cigarette smoking by 36.8% and current smoking of other tobacco by 38.1%. After adjusting for sociodemographic, school-level and environmental factors, 6.4% of the odds of current cigarette smoking and 8.9% of the odds of current smoking of other tobacco were explained by differences between schools.

## DISCUSSION

This study has five main findings. First, attending rural schools was significantly associated with increased odds of current smoking of cigarettes among students of fathers with high SES but not students of fathers with low SES. Second, attending rural schools was significantly associated with increased odds of current smoking of other tobacco products. Third, the association between fathers’ SES and current smoking of cigarettes differed by school location. In rural schools, students with high fathers’ SES were more likely to be current smokers but in urban schools, there was no association between father’s SES and current smoking. Fourth, environmental factors associated with current smoking of cigarettes and other smoked tobacco were similar, and included exposure to secondhand smoke and tobacco advertisements, having smoking peers or parents, and sale of cigarettes near schools. Being taught about tobacco in school predicted lower odds of smoking cigarettes but not smoking other tobacco products. Fifth, students of fathers with high SES were less likely to smoke other tobacco products compared to students of fathers with low SES.

The finding that a positive association between schooling in a rural area and adolescent cigarette smoking was much higher among high SES compared with low SES groups supports our proposition at the beginning of the study that the association between school location and adolescent tobacco use may be different at different levels of SES. It also suggests that students of high SES group are more vulnerable to cigarette smoking in rural compared to urban schools. Similar association was reported in a Scottish study although the result was not statistically significant perhaps due to reduced power caused by the introduction of many interaction parameters^19^. We also found that high SES was positively associated with cigarette smoking in rural schools but not in urban schools. These findings have important implications for tobacco control in Nigeria since the target populations for adolescent tobacco control programs may need to be different in different locations. Students with high parental SES need to be targeted in rural locations contrary to previous findings that low SES groups are at higher risk of tobacco smoking^10,11^.

Increased risk of tobacco smoking in rural compared to urban schools is consistent with findings from previous studies mostly in developed countries^7-9,20,21^. Possible contributory factors are higher exposure of students in urban schools to anti-tobacco messages and to tobacco teaching in school in this study. Increased knowledge about tobacco has been shown to reduce risk of adolescent smoking^22^. Conversely, Enugu State is not known for tobacco farming, so this may not explain the urban–rural differences in tobacco smoking in this study. Our finding that male students were more likely to smoke is consistent with previous studies in Nigeria and other countries^23-28^. Contrary to previous studies^29-31^, we found that tobacco smoking decreased with increasing age. A possible explanation is that these younger students were in the experimental phase of using tobacco. It could also be a pointer to the tobacco epidemic that is facing developing countries. Similar results were reported in rural Zambia^32^ and highlights the need for smoking prevention interventions that target younger students. The finding of increased odds of current smoking with increasing monetary allowance has been reported previously^33^; it suggests that it may be necessary to discourage monetary allowance to adolescents.

Several environmental factors were found to be associated with both types of tobacco smoking. Most notable were the influence of parental and peer smoking. Students who reported that all their friends or classmates smoked had 7 times and 4.5 times higher odds, respectively, of being current smokers of cigarettes compared to those with nonsmoking friends or classmates. Similar findings were reported in Iraq^34^. These observations could be due to peer selection whereby adolescent smokers tend to befriend other smokers. Another explanation could be that adolescent non-smokers tend to initiate smoking when they have smoking friends. The magnitude of the association was less with classmates’ smoking. These findings suggest the strong role of peer influence on adolescent tobacco smoking in this setting. Although it has been reported that adolescent smokers were likely to overestimate the smoking status of their friends^35^, this may not explain the finding in this study considering the magnitude of the association observed with peer smoking.

The finding that adolescents’ friends and classmates were significant predictors of tobacco use in this study makes it imperative that policymakers develop policies that decrease the ease with which young people obtain and supply tobacco. Possible strategies could include limiting the number of tobacco outlets, particularly around schools, and enforcing consistent and larger excise tax increases, making it harder for adolescents to afford, access, and supply tobacco. Similarly, the finding that students who had both smoking parents were twice as likely to currently smoke tobacco demonstrates the strong influence parents’ lifestyle has on their children, knowledge of which can be harnessed in adolescent tobacco control programs. Smoking prevention programs therefore need components focused on parents to help reduce adolescent smoking.

Despite the ban on pro-tobacco advertising in Nigeria^36^, a significant proportion of students (73%) in this study were exposed to some form of pro-tobacco advertisement in the past 30 days. Consequently, students who were exposed to pro-tobacco advertisements had increased odds of being current smokers. This finding emphasizes the need for more comprehensive bans on tobacco advertisements to minors. As legislations are not strictly enforced in Nigeria, implementation of plain packaging as part of a comprehensive approach to tobacco control could be worthwhile^37^. Similar findings were reported for the African region following a secondary analysis of the GYTS of 20 low- and middle-income countries^38^. Our finding that exposure to secondhand smoke in the past 7 days increased the likelihood that adolescents are current smokers is consistent with previous evidence^31^. These findings suggest that adolescents may perceive smoking as socially acceptable when they are consistently exposed to the behavior in their environment. In general, denormalization campaigns that reduce the attractiveness and accessibility of tobacco could decrease the social acceptability of smoking and make adolescents less likely to use tobacco. These denormalization strategies have been found to have population-wide effects that influence adolescents^39,40^. It is, therefore, critical that policy makers key into these strategies in Nigeria.

Students who could buy cigarettes near their school were markedly more likely to be current smokers compared with those who could not. This compares with findings in California and Canada, which showed higher prevalence of current smoking at schools located in neighborhoods with high tobacco outlet density compared to those without any tobacco outlets^41,42^. This finding presents the need for policy restricting tobacco sale around schools in Nigeria. We found that inclusion of tobacco in the school curriculum reduced the odds of current smoking of cigarettes by 43%, suggesting that this method of delivery of anti-tobacco messages may be effective. Similar findings were reported in a secondary analysis of GYTS in four South Asian countries^22^.

### Strengths and limitations

Our study has some notable strengths: it is the first study in Nigeria to examine predictors of adolescent tobacco use using multilevel analysis. It examined these predictors while investigating interaction that is often ignored in tobacco studies. In addition, the response rates were higher than the recommended 80% for global tobacco studies. The study also had high power (90%) to reliably test for associations. A few limitations are worth mentioning. The school-based design may limit generalizability of the study findings to all adolescents in Enugu, Nigeria. Nevertheless, adolescent school enrollment is high (71.1%) in this setting^43^ and a school survey is an effective way to collect data from adolescents. Additionally, students may over-report or underreport their tobacco use behaviors in self-administered surveys. We cannot determine the extent of this type of bias from this study, however, reliability studies in the US have shown good test-retest results for similar tobacco-related questions^44^. We measured SES using parents’ level of education. Although many studies on adolescent tobacco use have measured SES using more than one variable, parental education has been shown to be a reliable and stable measure of adolescent SES^45^. We did not collect data on tribe and religion; these are of potential relevance in the African setting and could be associated with tobacco use. Lastly, none of the associations observed can be established as causal because of the cross-sectional design of the study.

## CONCLUSIONS

We found an association between school location and current adolescent cigarette smoking that differed by socioeconomic status. This study also established that adolescent smoking of tobacco products other than cigarettes was higher in rural schools and among students of low SES. Our findings have demonstrated that exposure to secondhand smoke and tobacco advertisements, parental and peer smoking, and sale of tobacco around schools, were positively associated with adolescent tobacco smoking. Policy interventions to restrict access to tobacco products around schools, comprehensive bans on tobacco advertisements and sale to minors, in addition to behavioral and other context-specific and culturally adapted interventions are needed to bend the curve of increasing tobacco smoking in Sub-Saharan Africa. Further research is needed to better understand these country-specific interactions between geographical location, socioeconomic status and adolescent tobacco smoking.
